# Image-based deep learning in otology: a state-of-the-art review

**DOI:** 10.3389/fneur.2026.1884951

**Published:** 2026-08-03

**Authors:** Sining Wu, Yating Liao, Zhenhua Li

**Affiliations:** Hunan Provincial People's Hospital, Changsha, China

**Keywords:** artificial intelligence, deep learning, otology, otoscopy, temporal bone computed tomography

## Abstract

**Objective:**

Deep learning (DL), a core branch of artificial intelligence (AI), has revolutionized medical image analysis. Driven by advances in computational power and access to large-scale datasets, DL excels at extracting hierarchical features from complex, unstructured data. Accurate interpretation of imaging features is essential for diagnosing and treating middle and inner ear diseases. This narrative review aims to summarize current literature on the application of DL in otological imaging.

**Methods:**

A narrative literature search was conducted using the PubMed and MEDLINE databases. Keywords related to AI or DL in otology were used to identify relevant articles published up to the time of writing.

**Results:**

DL models have achieved favorable results in classifying common middle ear diseases (e.g., otitis media) using otoscopic images and in segmenting major inner ear structures (e.g., cochlea, ossicular chain) in 3D volumetric data. While 2D CNNs are mature for otoscopic classification, 3D U-Net and UNETR architectures dominate CT and MRI analysis. Models also show value in low-dose CT reconstruction and multimodal diagnosis.

**Conclusion:**

DL has demonstrated strong potential to improve clinical decision-making and healthcare efficiency in otology. However, the field faces challenges related to data scarcity for rare diseases, poor segmentation performance for tiny structures (e.g., stapes), and a lack of integration with clinical workflows. Future efforts should focus on standardizing data and optimizing network structures for specific modalities.

## 1 Introduction

Deep learning (DL) (1), a transformative subset of artificial intelligence (AI), uses multilayered neural networks to automatically learn complex patterns. Since 2012, DL has outperformed traditional methods in tasks such as image classification and speech recognition, leading to its rapid adoption in medical image analysis. By assisting clinicians in saving time, reducing manual labor, and minimizing diagnostic errors, AI-based computer-aided diagnosis (CAD) systems play a crucial role in lesion detection and treatment evaluation.

More than 90% of healthcare data comprises medical imaging data. Common imaging modalities include magnetic resonance imaging (MRI), X-ray radiographs, computed tomography (CT), ultrasound, positron emission tomography, endoscopic images or videos, and histopathological slide images, among others. The application of AI in medical imaging encompasses five major tasks: image reconstruction, lesion detection, image segmentation, image registration, and CAD. The core concept of AI-based analysis of medical images is to interpret and learn from imaging data through algorithmic approaches. This process typically begins with the acquisition of large volumes of high-quality image data, followed by preprocessing. Subsequently, various deep neural network architectures are employed to extract features at multiple levels, enabling end-to-end automatic feature learning through a series of nonlinear transformations. Ultimately, this facilitates the extraction of meaningful information from images for analytical and predictive purposes. In clinical practice, numerous CAD systems already assist physicians in disease diagnosis, primarily focusing on organs and regions such as the lungs (2), breasts (3), heart (4), and brain (5), hereby supporting more accurate diagnosis, treatment planning, and prognostic evaluation.

Conventional imaging modalities in otology include endoscopy, temporal bone CT, and MRI. Endoscopy is primarily used to visualize the external auditory canal and tympanic membrane. CT provides excellent visualization of bony structures within the temporal bone, whereas MRI offers superior soft-tissue contrast, making it advantageous for assessing the membranous labyrinth, nerves within the internal auditory canal, and temporal bone tumors. The intelligent processing of otological images poses significant challenges due to the intricate and tightly coupled anatomical structures within the temporal bone, compounded by its exceptionally small imaging field of view. DL applications in this field primarily focus on disease classification, lesion detection, and segmentation of anatomical landmarks or pathological regions. These applications support tasks such as binary or multiclass disease categorization, lesion localization or identification, and segmentation of anatomical structures or lesions. The main imaging modalities involved are endoscopic images, temporal bone CT, and MRI. Currently, single-modality imaging remains the most prevalent approach in clinical otological imaging research, with studies predominantly concentrating on the diagnosis of middle and inner ear diseases.

This review summarizes recent advances in DL for otological imaging, categorized by the three primary modalities: endoscopic images, temporal bone CT, and MRI.

## 2 Endoscopic images

Endoscopic examination is a routine procedure in otology. Accurate endoscopic diagnosis facilitates early detection, precise diagnosis, and longitudinal monitoring of both external and middle ear diseases. Endoscopic images provide direct visualization of the external auditory canal and tympanic membrane, offering immediate evidence of pathologies in these regions as well as indirect clues to certain middle ear conditions. Consequently, they constitute a crucial diagnostic basis for diseases affecting the external and middle ear. The primary endoscopic techniques include otoscopy and video otoscopy. Currently, AI applications based on endoscopic images predominantly focus on the identification and classification of common external and middle ear diseases, such as otitis media with effusion, chronic suppurative otitis media, acute otitis media, and cerumen impaction. In these studies, research objectives encompass both binary classification (6–11) and multiclass classification. Representative studies on multiclass classification and their methodological characteristics are summarized in Table 1. Notably, the diagnosis of otitis media with effusion is the most frequently addressed task within multiclass classification frameworks.

**Table 1:** Studies on classification of otoscopic images.

Author	Year	Number of images	Number of classes	Model	Class descriptions	Outcomes (ACC)
Livingstone et al. (66)	2019	529	3	CNN	Normal, Tympanostomy tube, Cerumen impaction	84.40%
Sundgaard et al. (67)	2022	1,336	3	Inception V3	AOM, OME, Normal	85%
Alhudhai et al. (68)	2021	857	4	CNN	Normal, AOM, CSOM, Cerumen impaction	98.26%
Wu et al. (69)	2020	12,203	3	Xception MobileNetV2	Normal, AOM, OME	97.45% 95.72%
Chen et al. (70)	2022	2,820	10	Inception V3	Normal, AOM, AC, CSOM, OME, Tympanic membrane perforation, Cerumen impaction, Tympanostomy tube, Tympanic membrane retraction, Otomycosis	98%
Mao et al. (71)	2022	2,657	4	EfficientNetB6	Otomycosis, Cerumen impaction, Otitis externa, Normal ear canal	92.42%
Crowson et al. (72)	2022	639	3	ResNet-34	Normal, AOM, OME	80.80%
Viscaino et al. (73)	2022	22,000	4	VGG-16	COM, Cerumen impaction, OME, Normal	92%
Zhong et al. (74)	2024	20,122	3	ZFNet TSL16	AOM, OME, Normal	97.87% 97.62%
Hee et al. (75)	2025	2,964	4	ConvNeXt	Normal, AOM, OME, COM	88.7%
Guo et al. (76)	2025	819	4	VGGNet-19	Normal, AOM, OME, COM	94.51%
Elmorsy et al. (77)	2025	282	6	MobileNet DenseNet169	AOM, CSOM, Earwax, Miringoskleroz, Normal, Otitis externa	99.54%
Bryton et al. (78)	2025	296	3	ResNet-18	Patent tube in place, Tube extruded into canal, Tube absent	97.10%
Sruthi et al. (79)	2025	1,139	4	ResNet-18	AOM, OME, no effusion or infection, no TM	92.5%
Chien-Yi et al. (80)	2026	607	3	U-Net	COM, OME, Normal	96.76%
Feiyan et al. (81)	2026	24,233	9	Best-EarNet	AOM, CME, CSOM, EACB, IC, NE, OE, SOM, TMC	92.14%

ACC, accuracy; AOM, acute otitis media; CSOM, chronic suppurative otitis media; OME, otitis media with effusion; COM, chronic otitis media; AC, acute myringitis; CNN, convolutional neural network.

In general, the greater the number of disease categories included in a study, the larger the volume of training data required. Because DL models are highly dependent on large-scale datasets, achieving automated diagnosis for relatively rare conditions, such as tumors of the external auditory canal and middle ear, remains challenging. Methodologically, most studies train classifiers directly on entire endoscopic images. Alternatively, to reduce background interference and enhance model performance and efficiency, some studies adopt a two-stage strategy involving initial segmentation of key anatomical structures (e.g., the tympanic membrane), followed by subsequent model training. To augment sample size, data augmentation during preprocessing is commonly used; synthetic data generation using neural networks has also been employed to expand datasets (8, 12). Beyond static examination images, some studies have utilized intraoperative endoscopic video (11) or examination videos (13, 14) for training. By analyzing morphological changes in the tympanic membrane across different states in videos, such approaches can help determine the presence of conductive hearing loss (15). To enhance model robustness and generalizability in response to practical challenges, such as variations in otoscope models and substantial heterogeneity in image quality across medical institutions, the use of multicenter datasets for joint training has become a growing trend in recent research (7, 16). Concurrently with the development of AI diagnostic models, various novel tele-otoscopy devices have emerged (17, 18). These systems connect otoscopes to smartphones for image acquisition and integrate AI algorithms for automated analysis. This integration facilitates screening for otitis media, supports post-tympanostomy tube follow-up, and assists in determining whether ventilation tubes remain properly positioned (19). To enhance diagnostic accuracy, given that otoscopic images alone may not fully capture middle ear status, some researchers have pursued multimodal fusion by integrating otoscopy with pure-tone audiometry or temporal bone CT, in line with the growing use of multimodal approaches in medical imaging. For instance, Noda et al. (20) employed the GPT-4 V model, using 190 otoscopic images combined with patient demographics (age, sex) and chief symptoms for four-class diagnosis. Taleb et al. (21) performed segmentation of the tympanic membrane and malleus handle by fusing otoscopic images with temporal bone CT scans, applying DL algorithms for multimodal fusion to facilitate preoperative image registration.

## 3 Ct

Temporal bone CT is a routine examination for the diagnosis and management of middle and inner ear diseases. Its primary advantage lies in its excellent resolution of bony structures, which facilitates disease diagnosis, delineation of anatomical and pathological relationships preoperatively, surgical planning, and postoperative outcome evaluation. The intelligent processing of temporal bone CT images presents significant challenges due to several factors. For example, the intricate and tightly coupled anatomy within a small imaging field often results in limited resolution and high variability in shape and texture features. Additional difficulties arise from the lack of standardized imaging protocols across institutions, class imbalance in sample distributions, and the scarcity of high-quality annotated datasets. Currently, the majority of studies focus on the analysis of conventional high-resolution temporal bone CT, which represents the most common clinical scanning protocol. However, research using micro-CT (22, 23) and cone-beam CT (24, 25) has also been reported. Research in this area primarily encompasses automatic segmentation of anatomical landmarks and lesions, classification and diagnosis of middle ear diseases, and enhancement of temporal bone CT image quality.

### 3.1 Automatic segmentation

In temporal bone CT, key anatomical landmarks of high clinical relevance include the ossicular chain, facial nerve, inner ear, and sigmoid sinus. Accurate identification of these structures is crucial for assessing lesion extent, selecting surgical approaches, and evaluating surgical complexity. Cochlear segmentation, for instance, can guide electrode selection in cochlear implantation and holds significant value in robotic-assisted surgery (26). Furthermore, segmentation can support otological clinical education, surgical skills training, and the professional development of otologists.

Automatic segmentation of these critical anatomical structures presents a formidable challenge. This is due to their small size (some occupying less than 1% of the target area in a single CT slice); substantial heterogeneity in shape, size, and density; and often poorly defined boundaries between the target region and surrounding tissues. With advances in DL algorithms and model architectures, research has evolved from region-of-interest detection (27, 28) to precise segmentation tasks (23). The scope has expanded from segmenting single landmarks (27, 29, 30) to multiple landmarks simultaneously (31–33), and from 2D (27, 34, 35) to 3D segmentation (32). Early studies predominantly employed 2D convolutional neural networks (CNNs). However, for high-resolution CT (HRCT) images of the temporal bone, interslice correlations are significant. Conventional 2D CNNs fail to fully leverage the inherent continuity of temporal bone HRCT images. Because accurate assessment of complex pathologies within intricate middle ear structures relies on this sequential information, 3D CNNs capable of processing entire image volumes have progressively become the predominant approach for temporal bone HRCT analysis. Most segmentation models are based on modified U-Net architectures, although a few studies have used UNETR to segment structures such as the cochlea (30) and ossicular chain (31). Existing literature indicates that automatic segmentation achieves higher performance for larger bony structures (e.g., inner ear, cochlea) than for soft tissues such as the facial nerve. Performance is particularly low for the ossicles, especially the stapes, because of their poor contrast in CT images and the considerable manual annotation error associated with them. Subsequently, multiple studies have used enhanced 3D U-Net architectures for the segmentation of cone-beam CT (25), temporal bone micro-CT (22, 23), and HRCT (32, 36) images. These architectural enhancements frequently incorporate modules such as attention mechanisms, residual blocks, dense connections, and dilated convolutions. In parallel, approaches such as refinement of activation functions and augmentation of training datasets have been implemented to improve performance and enhance model robustness (26). Dilated convolutional blocks can extract target features at multiple scales, whereas residual multi-kernel pooling blocks use several pooling kernels of different sizes in parallel to detect targets of varying dimensions. A summary of key 3D segmentation studies is provided in Table 2.

**Table 2:** Studies on segmentation of temporal bone images.

Author	Year	Number of samples	Segmentation targets	Model	Outcomes (DSC)
Li et al. (32)	2020	64	Malleus, Incus, ECC, ICC, LSC, PSC, SSC, Vestibule, IAM	3D-DSD Net	0.7718
Lv et al. (82)	2021	30	Cochlea, LA, OC, FN	W-Net	0.90, 0.85, 0.77
Jiang et al. (83)	2021	39	OC, FN, LA	W-Net	0.855, 0.91, 0.703
Wang et al. (65)	2022	158	Malleus, Incus, Stapes	3D VNet	0.92, 0.925, 0.835
Ke et al. (84)	2023	80	FN, OC, IE, Cochlea, Vestibule, LSC, SPSC, ICA, IAC, JB, EAC	W-Net	0.746, 0.891, 0.912, 0.897, 0.862, 0.843, 0.842, 0.854, 0.868, 0.714, 0.859
Li et al. (30)	2023	92	Cochlea	UNETR	0.92
Zhou et al. (31)	2023	147	OC, IAC, FN, LA	UNETR	0.8121, 0.8809, 0.6858, 0.9329
Li et al. (85)	2025	271	Cochlea	SegResNet	0.94
Xie et al. (86)	2025	304	OC, LA	SemiEBS	0.979

DSC, Dice similarity coefficient; LSC, lateral semicircular canal; SPSC, superior and posterior semicircular canals; ICA, internal carotid artery; JB, jugular bulb; OC, ossicular chain; IAC, internal auditory canal; FN, facial nerve; EAC, external auditory canal; ECC, external contour of cochlea; ICC, internal cavity of cochlea; PSC, posterior semicircular canal; SSC, superior semicircular canal; IAM, internal acoustic meatus; LA, labyrinth; IE, inner ear.

### 3.2 Disease diagnosis

Temporal bone CT plays a critical role in the diagnosis of otological diseases, preoperative assessment, and surgical planning because of its ability to visualize osseous structures and local pathologies. Classification and diagnosis of otological conditions using temporal bone CT typically rely on modified CNN models. Existing research covers a range of applications, including the diagnosis of otosclerosis (37–39), inner ear malformations (40), cochlear abnormalities (41), middle ear cholesteatoma (28, 42–44), and identification of a high-riding jugular bulb (45). Most of these studies are based on single-center data, although a few have adopted multicenter designs (43). Key publications in this area are summarized in Table 3. Representative studies include the work of Fujima et al. (38), who proposed a two-stage approach for diagnosing otosclerosis. Their method involves initial rectangular region-of-interest detection around the vestibular window in 2D CT images, followed by classification. Eroğlu et al. (42) extracted imaging features using three different DL network architectures and combined them with a support vector machine classifier to distinguish between chronic suppurative otitis media and cholesteatomatous otitis media on temporal bone CT. In2024, Qi et al. (44) analyzed 1,019 temporal bone CT scans (410 with chronic suppurative otitis media, 398 with middle ear cholesteatoma, and 211 normal cases) with coarse-grained contour boundary annotations. Their methodology involved pretraining and focal segmentation using the nnU-Net model with coarse-grained focal segmentation labels, followed by three-class disease classification.

**Table 3:** Studies on classification of temporal bone CT images.

Author	Year	Number of samples	Image type for model	Class descriptions	Model	Outcomes (ACC)
Wang et al. (28)	2020	1,147	2D	Normal, CSOM, Cholesteatoma	Faster R-CNN	76.7%
Fujima et al. (38)	2021	198	2D	Normal, Otosclerosis	AlexNet, VGGNet, GoogLeNet, ResNet	89, 72, 81, 86, 86%
Tan et al. (39)	2021	1,338	2D	Normal, Otosclerosis	VGG-19	98.07%
Eroğlu et al. (42)	2022	200	2D	Cholesteatoma, CSOM	AlexNet, GoogLenet, DenseNet201 + SVM	95.4%
Ogawa et al. (40)	2022	6,776	3D	Normal, Inner ear malformation	3D-VAE	95.6%
Duan et al. (87)	2022	119	2D	Cholesteatoma, Langerhans cell histiocytosis, CSOM	VGG16-BN	98.7%
Takahashi et al. (88)	2022	164	2D	Cholesteatoma with or without mastoid extension	MobileNetV2	81.14%
Li et al. (41)	2023	165	2D	Normal, Cochlear malformation	ResNet10, ResNet50, SE-ResNet50, DenseNet121	91, 94, 93, 73%
Chen et al. (43)	2024	1890	3D	Normal, Cholesteatoma, CSOM	CNN	81.8%
Qi et al. (44)	2024	158	2D	Normal, CSOM, Cholesteatoma	NnuNet	92.33%
Hasan et al. (45)	2025	600	2D	Normal, High-riding jugular bulb	CNN	94.8%
Li et al. (62)	2026	373	3D	Normal, cochlear malformation	DenseNet121	93%

ACC, accuracy; CSOM, chronic suppurative otitis media; CNN, convolutional neural network; SVM, support vector machine.

### 3.3 Image quality enhancement

CT image quality directly impacts the diagnosis and treatment of otological diseases. Low-dose CT protocols often result in insufficient resolution, pronounced artifacts, and low signal-to-noise ratios. However, given the proximity of the ear to radiation-sensitive structures such as the eyes, thyroid gland, and central nervous system, high-dose scans increase the risk of radiation-induced damage, including teratogenic or carcinogenic effects, cataracts, and thyroid cancer. Consequently, several studies have leveraged DL techniques for intelligent processing of low-resolution CT images, aiming to reduce radiation exposure while enhancing image quality. Various postprocessing methods, including denoising, contrast enhancement, and reconstruction, can effectively mitigate image noise, reduce irrelevant information, and improve overall image quality and visualization. These enhancements facilitate delineation of pathological regions and critical anatomical structures. For instance, Fujita et al. (46) and Brockstedt et al. (47) used DL algorithms to enhance the resolution of temporal bone CT, thereby improving diagnostic accuracy for middle ear diseases. Wang et al. (48) and Boubaker et al. (49) combined temporal bone CT with DL approaches to reduce radiation dose while maintaining diagnostic image quality. Beysan et al. (50) evaluated the utility of DL algorithms in improving assessment of stapes and tympanic nerve anatomy. Similarly, Xiaoguang et al. (51) used a DL method to automatically align temporal bone CT images, using the lateral semicircular canal as a reference standard.

Notably, anatomical segmentation, disease diagnosis and image quality enhancement for temporal bone CT are not isolated research directions but closely interconnected technical modules. Precise segmentation of temporal bone structures provides standardized region-of-interest inputs to improve the robustness of disease classification models, while DL-driven image denoising and low-dose reconstruction fundamentally resolve the performance bottlenecks of segmentation and lesion detection caused by noisy low-quality CT scans. In turn, clinical demands for identifying minute otological lesions continuously guide the iterative upgrade of image enhancement pipelines and multi-structure segmentation architectures.

## 4 MRI

As the radiation-free soft-tissue imaging modality for inner ear lesions, MRI relies on 3D volumetric sequences to assess Meniere’s disease, vestibular schwannoma and cochlear implantation candidates, distinct from bony-focused temporal bone CT. Existing deep learning (DL) studies for otologic MRI fall into three interconnected directions. For membranous labyrinth segmentation, 3D U-Net, V-Net, CCFT 3D U-Net and Swin UNETR achieve Dice coefficients above 0.88 for large fluid spaces (52–57), yet micro vestibular schwannoma segmentation only reaches DSC = 0.61 due to scarce small-lesion samples and cross-scanner signal variation (58), and most networks only support single-task delineation instead of joint anatomy-lesion identification. In disease classification, few models integrate MRI with audiometric or CT multimodal information (59). For scan optimization, DL reconstruction shortens acquisition time and mitigates motion artifacts in routine cases, but fails to adapt to malformed or post-implant inner ears (60, 61). A summary is provided in Table 4.

**Table 4:** Studies on MRI images.

Author	Year	Samples	Tasks	Model	Outcomes(DSC)
Jeevakala et al. (56)	2020	50	Internal auditory canal segmentation	Mask R-CNN + U-Net	94%
Vaidyanathan et al. (52)	2021	944	Membranous labyrinth segmentation	3D U-Net	88%
Ahmadi et al. (54)	2022	179	Membranous Labyrinth segmentation	V-Net	90.0%
Suresh et al. (89)	2024	252	Vestibular Schwannoma segmentation	nnU-Net	61%
Yoo et al. (53)	2025	129	Inner ear segmentation	CCFT 3D U-Net	94.26%
Zheng Wang et al. (57)	2026	259	Vestibule segmentation classification	U-Net, YOLOv5s	96.4% 87.7%
Wooseung Kim et al. (55)	2026	82	Membranous labyrinth segmentation	Swin UNETR	91.9%

## 5 Prospects and challenges

Deep learning has achieved progressive achievements in the analysis of otoscopic images, temporal bone CT and inner ear MRI, the three mainstream imaging modalities for otology. Existing studies have developed diverse algorithms for disease classification, anatomical segmentation and image reconstruction, yet the current field still faces multiple practical and technical obstacles combined with the limitations reflected in published literature.

From the perspective of data construction, datasets remain the primary bottleneck restricting model performance. As summarized in Tables 1–4, most current studies rely on single-center retrospective data. For common otological diseases such as acute otitis media (AOM), otitis media with effusion (OME) and chronic suppurative otitis media (CSM), researchers have collected thousands of images and acquired satisfying classification accuracy (over 80%). However, for rare lesions including external auditory canal tumors and inner ear malformations, valid annotated samples are extremely insufficient (62). Even with data augmentation and synthetic image generation (58), it is still difficult to eliminate class imbalance. In addition, otological images feature tiny anatomical structures such as the stapes and semicircular canals. Manual annotation requires professional otologists and radiologists, which brings inevitable inter-rater deviation. Heterogeneous imaging parameters across different devices and institutions further aggravate dataset variability, making single-center models prone to overfitting and poor external generalization. Strict medical privacy rules also hinder multi-center data sharing and joint research.

In terms of algorithm design and model performance, the defects of existing networks are closely related to their application scenarios across different imaging modalities. For otoscopic images, most classification models adopt 2D CNNs such as VGG, ResNet and Inception. Although two-stage strategies combining pre-segmentation and classification have been proposed to reduce background interference, most models can only complete single classification tasks (63). Some studies have fused otoscopy with pure-tone audiometry or CT (20, 64), but the fusion frameworks are relatively simple and fail to fully excavate complementary information between multimodal data. For temporal bone CT, 2D CNNs were initially widely used, while 3D U-Net, UNETR and other 3D architectures have gradually become mainstream for volumetric data analysis (31, 32, 62). According to the segmentation results in Table 2, large bony structures (cochlea, labyrinth) achieve a high Dice coefficient, yet the segmentation of tiny soft tissues and ossicles (especially stapes) still performs poorly (Dice < 0.835) due to low tissue contrast (65). Besides, multiple studies have adopted DL for low-dose CT reconstruction to reduce radiation exposure (49, 50), but there is still room for improvement in balancing image quality and radiation dose. For inner ear MRI, mainstream models including 3D U-Net and V-Net focus on membranous labyrinth segmentation. The segmentation of vestibular schwannoma shows unsatisfactory results (Dice = 61%) (58), indicating that existing algorithms struggle with small tumors and subtle lesions. Moreover, most models are designed for single tasks, lacking interpretability, and 3D networks require massive computing resources, which conflicts with the deployment demands of lightweight bedside devices.

Clinically, the disconnection between AI models and routine workflows is prominent. Current research prototypes are rarely compatible with hospital PACS systems. There are no unified industry specifications for model application, operation norms and responsibility division, which hinders large-scale clinical promotion. Additionally, the whole industrial chain of otological AI faces high costs in data annotation, model training, equipment deployment and daily maintenance, and the absence of supporting commercial and reimbursement policies further discourages grassroots medical institutions from adopting relevant technologies.

Targeted solutions can be proposed based on the deficiencies of existing literature and verified technical routes. First, to address data shortages and heterogeneity, standardize imaging and annotation protocols, and adopt transfer learning, few-shot learning and federated learning to make full use of existing single-center data without data leakage. Generative adversarial networks (GAN) can continue to be applied to generate synthetic otoscopic and CT images (12), so as to supplement samples of rare diseases. Second, optimize directional network architectures for different imaging modalities: for otoscopy, explore advanced multimodal fusion frameworks combining images, audiometric data and clinical information (20, 64); for CT and MRI, embed attention mechanisms and dilated convolutions into 3D networks (65) to enhance the recognition of tiny structures and subtle lesions, and develop lightweight, interpretable models for clinical deployment. Third, further promote DL-based CT reconstruction algorithms to optimize radiation protection schemes. Fourth, carry out large-scale multi-center prospective validation for existing models, and formulate unified application standards and industry norms. In the long run, deep learning will be deeply integrated into otological screening, preoperative planning, intraoperative navigation and postoperative evaluation, driving the overall intelligent upgrading of otological imaging.

## 6 Conclusion

This review systematically summarizes deep learning applications in otoscopic imaging, temporal bone CT and inner MRI, covering disease classification, anatomical segmentation, lesion detection and image quality enhancement. Current DL models have achieved favorable results in the diagnosis of common middle ear diseases and segmentation of major inner ear structures. As shown in the included studies and tables, 2D CNNs are mature for otoscopic image classification, and 3D network architectures represented by U-Net and UNETR have become the mainstream for 3D temporal bone and inner ear volumetric data analysis. Relevant models have also shown practical value in low-dose CT reconstruction and multimodal auxiliary diagnosis.

Nevertheless, the field is still in the initial stage of clinical translation. Restricted by insufficient and imbalanced datasets, unsatisfactory segmentation performance of tiny structures, insufficient multimodal fusion capability and high deployment costs, most existing research results remain in the experimental stage. The future development of otological imaging DL requires joint efforts in multiple aspects: standardizing multi-center data and annotation specifications, adopting few-shot learning and synthetic data technology to supplement rare disease samples; optimizing modal-specific network structures to improve the segmentation accuracy of micro-lesions and model interpretability; promoting the docking between AI systems and hospital clinical platforms; and completing multi-center external validation.

With the continuous optimization of algorithms, data systems and clinical supporting norms, deep learning will not only serve as an efficient auxiliary tool for otological diagnosis and surgical planning, but also play an important role in clinical teaching and surgical robot research, ultimately improving the diagnosis and treatment outcomes of patients with ear diseases.
